# Predictors of the onset of low handgrip strength in Europe: a longitudinal study of 42,183 older adults from 15 countries

**DOI:** 10.1007/s40520-024-02800-z

**Published:** 2024-08-07

**Authors:** Rizwan Qaisar, M. Azhar Hussain, Fabio Franzese, Asima Karim, Firdos Ahmad, Atif Awad, Abeer A. Al-Masri, Shaea A. Alkahtani

**Affiliations:** 1 Basic Medical Sciences, College of Medicine, University of Sharjah, 27272 Sharjah, United Arab Emirates; 2 Space Medicine Research Group, Research Institute for Medical and Health Sciences, University of Sharjah, 27272 Sharjah, United Arab Emirates; 3 Cardiovascular Research Group, Research Institute for Medical and Health Sciences, University of Sharjah, 27272 Sharjah, United Arab Emirates; 4 Department of Finance and Economics, College of Business Administration, University of Sharjah, 27272 Sharjah, United Arab Emirates; 5 Department of Social Sciences and Business, Roskilde University, DK-4000 Roskilde, Denmark; 6 SHARE Berlin Institute, Chausseestraße 111, 10115 Berlin, Germany; 7 Department of Physiology, College of Medicine, King Saud University, 11451 Riyadh, Saudi Arabia; 8 Exercise Physiology Department, College of Sport Sciences and Physical Activity, King Saud University, 11451 Riyadh, Saudi Arabia

**Keywords:** Handgrip strength, Risk factors, Quality of life, SHARE data

## Abstract

**Objectives:**

A low handgrip strength (HGS) is a significant risk factor for multiple diseases. However, most relevant studies investigate the complications of a low HGS, while the risk potential of causative factors of low HGS remain poorly characterized.

**Methods:**

We investigated the potentials of quality of life, depression, dyslipidaemia, diabetes mellitus, cancer, Alzheimer’s disease, stroke, frailty, and difficulties performing daily activities in predicting low HGS (≤ 27 kg for men, ≤ 16 kg for women) in European older adults aged 50 or above from 15 countries (n = 42,183). All data was collected from four successive waves of survey of health, ageing, and retirement in Europe (SHARE) conducted between 2013 and 2020. Logistic models are applied, and estimated effects are presented as odds ratios and probabilities.

**Results:**

Collectively, 3016 participants (men; n = 1395; 7.38%, women; n = 1621, 6.97%) developed low HGS during the 6.5 years study period. After adjusting for covariables, we identified an advancing age (1.6–48.1% points higher risk of low HGS), male gender (1.0%-point higher risk of low HGS), lower quality of life (1.6%-point higher), and stroke (1.5%-points) as significant risk factors for low HGS. We also found a dose-dependent association of Euro-D depression scores with the risk of low HGS, as the higher scores were associated with between 0.6- and 2.3%-points higher risk of developing low HGS than participants without depression. Among physical performance indicators, difficulty climbing stairs (2.0%-points higher low HGS risk) or rising from a chair (0.7%-points) were significantly associated with developing low HGS. Lastly, frailty (0.9%-points higher risk of low HGS) and the fear of falling down (1.6%-points higher risk) also increased the risk of developing low HGS.

**Conclusion:**

Altogether, we report several risk factors for developing low HGS. Our observations may help evaluating and monitoring high-risk population for developing low HGS in pre-clinical settings.

## Introduction

The skeletal muscle is the largest organ in the human body and plays a pivotal role in the health and maintenance of multiple body systems [1]. It also maintains a functional interface with other body systems, including skeletal, cardiovascular, nervous, gastrointestinal, immune, and excretory systems [2]. Thus, a bidirectional crosstalk exists so that a defect in these body systems negatively affects skeletal muscle and vice versa [2]. Thus, it may be critical to timely identify and treat a defect in skeletal muscle.

Skeletal muscle exhibits a remarkable plasticity in various disease conditions. However, the type, severity, and exposure time of specific diseases can eventually lead to a defect in the skeletal muscle adaptation process [2]. Two common manifestations of skeletal muscle defect are muscle wasting and weakness [3]. In most cases, muscle weakness may precede muscle wasting during ageing and maybe a more substantial risk factor for a dependent lifestyle [4]. Thus, it may be imperative to monitor muscle force-generating capacity before a more established degree of muscle pathology develops.

Handgrip strength (HGS) is a simple and objective muscle strength measurement. The normal HGS in a healthy person is affected by several physiological factors, including age, gender, ethnicity, diet, and geographical factors. Therefore, normative values of HGS are recognized for various age groups, both genders, and geographical regions [5]. A reduced HGS is a signature finding in age-related muscle loss, termed sarcopenia [1]. A low HGS is also considered the primary sign of probable sarcopenia before the diagnosis is established with low muscle mass and/or physical capacity [6]. The European Working Group on Sarcopenia in Old People (EWGSOP) defines a low HGS as ≤ 27 kg for men and ≤ 16 kg for women [6]. The global prevalence of sarcopenia is progressively increasing, emphasizing the need for timely monitoring and identifying low HGS [7]. This increase is partly due to an increasing lifespan. However, unhealthy lifestyle, including physical inactivity, unhealthy food, environmental pollution, and concomitant diseases are also considered important contributors to an increasing prevalence of sarcopenia [7–9].

In recent years, there has been a significant increase in studies investigating the consequences of low HGS on generalized health [1, 2]. It is now recognized that low HGS is an independent risk factor for several diseases of metabolic, degenerative, and inflammatory nature [2]. Further, a low HGS increases the risk of a dependent lifestyle in geriatric adults. Specifically, older adults with low HGS exhibit a reduction in ambulatory capacity, quality of life, and activities of daily living [2]. Based on these findings, HGS is also proposed as a new vital sign of health in older adults [10]. Consequently, HGS is associated with several health-related metrics. For example, patients with low HGS demonstrate increased risks of hospitalization, functional disabilities, poor psychological health, and mortality [10]. Together, these consequences support the occurrence of higher mortality in older adults with low HGS [2]. Thus, these observations necessitate the timely monitoring and identification of risk factors associated with low HGS.

Most relevant studies investigate the associations of low HGS with future onset of morbidity and mortality due to various diseases [2]. Conversely, the associations of common diseases and lifestyle factors with the future development of low HGS remain poorly characterized. Several common diseases, such as diabetes mellitus, stroke, Alzheimer’s disease, hypertension, and osteoarthritis, can increase the risk of developing low HGS [11]. Older adults with poor quality of life and cognitive decline are also more likely to develop low HGS [11, 12]. Lastly, difficulty performing activities of daily living, such as rising from a chair, climbing stairs, or dressing oneself, can be independent risk factors for developing low HGS [11]. A defect in these activities suggests poor functioning of lower limb muscles, which can be an early indication of generalized muscle weakness, including a low HGS. Therefore, these activities may hold predictive potential for a low HGS. It is critical to thoroughly characterize these factors of daily living and common diseases for their potential to predict low HGS. However, the relevant studies are scarce and involve small datasets. Conversely, a large relevant study involving population from a whole continent remains elusive. We aimed to characterize the associations of these factors with the risk of developing low HGS in older adults. Several attributes establish the novelty and biological relevance of our study. First, unlike most other studies, we investigated older adults with normal HGS at baseline and investigated the future onset of low HGS relevant to several diseases and lifestyle factors. Second, several diseases or lifestyle factors work in conjunction to negatively affect skeletal muscle [3]. However, most previous studies have overlooked the confounding effects of concomitant factors on HGS. We overcame this problem by statistically adjusting for comorbidities and confounders while characterizing the prospective associations of individual risk factors with low HGS. Lastly, most relevant data stems from localized communities and small sample size, with limited relevance to the global population pool [1, 4, 11]. Conversely, we investigated a large sample size of 42,183 older adults from 15 representative countries of the European continent.

This study aims to characterize the risk factor potential of various cognitive, metabolic, and physical comorbidities with the future onset of low HGS among older European adults. We used the standardized survey of health, ageing, and retirement in Europe (SHARE) dataset for this study [13]. We hope that our observations will characterize the high-risk population for developing low HGS and associated disorders and assist the policymakers in improving the health span and quality of life of the geriatric population.

## Materials and methods

The SHARE dataset is a harmonized panel survey spanning multiple European countries, targeting individuals aged 50 years and older [14]. The data collection was conducted by computer-assisted personal interviews encompassing various domains, such as demography, socioeconomic factors, living conditions, and both physical and mental health. The baseline data, constituting the background characteristics, were drawn from wave 5 of SHARE, conducted in 2013. Subsequent waves (6, 7, and 8) conducted in 2015, 2017, and 2019/2020, respectively, provided follow-up information on HGS. The sample includes a total of all 15 countries available in SHARE wave 5, including Austria, Germany, Sweden, Netherlands, Spain, Italy, France, Denmark, Switzerland, Belgium, Israel, Czech Republic, Luxembourg, Slovenia, and Estonia.

The assessment of HGS was executed using a hand-held dynamometer (Smedley, S Dynamometer, TTM, Tokyo, 100 kg). The interviewer demonstrated the procedure before asking the respondent for willingness to perform the test. Medical exclusion criteria were swelling or inflammation, severe pain or recent injury, and recent surgery to the hand. Respondents were instructed to press the dynamometer with both their left and right hands, each repetition performed twice with alternations between the hands. If respondent had problem with one hand, measurements were only taken with the other hand. The test was performed with the respondent standing upright, the upper arm parallel to the upper body and the lower arm at a 90-degree angle to the upper arm. The test could be performed also in a sitting position if necessary. Interviewers were trained for the grip strength test based on harmonized training. A study on interviewer effects in SHARE revealed that 5–8% of the variance in HGS measure is related to the interviewers [15]. The highest recorded value from these four measurements were employed in the subsequent analysis [16]. For the purpose of this study, a low HGS was defined based on gender-specific thresholds, following the guidelines established by the European Working Group on Sarcopenia in Older People (EWGSOP2), with a threshold of 27 kg for men and 16 kg for women [6].

The SHARE wave 5 was used to derive all covariates. Quality of life was assessed using a composite index covering the dimensions of control, autonomy, self-realization, and pleasure (CASP-12) [17]. This index consisted of 12 items, with each of the four subcategories featuring three questions. Respondents were asked to rate how often they experienced specific feelings or thoughts using the response options “often,” “sometimes,” “rarely,” or “never.” These responses were scored as 1 (often), 2, 3, and 4 (never). The CASP-12 composite index, comprising the sum of the scores from the 12 indicators, has a range from 12 (indicating minimum well-being) to 48 (maximum well-being). The index was subsequently categorized into three groups for analysis purposes, spanning score ranges of 12–24 (low well-being), 25–36 (medium well-being), and 37–48 (high well-being) [17].

Mental health was assessed utilizing the Euro-D depression scale, which is an additive composite index based on the number of reported depressive symptoms. This index consisted of 12 symptoms, including sadness, having no hopes for the future, sleep disturbances, feelings of guilt, irritability, and loss of appetite [17]. Scores on the Euro-D scale ranged from 0 to 12, with higher scores indicating a greater number of depressive symptoms. For our analysis, Euro-D scores were categorized into four groups: 0 (no depression), 1–3, 4–6, and 7–12 (highest level of depression) [17].

The presence of comorbidities was determined by the respondents’ reporting of high blood pressure, high blood cholesterol, diabetes mellitus or high blood sugar, cancer, Alzheimer’s disease, osteoarthritis, and stroke. This information was collected through a list of diseases presented to the respondents, who indicated whether they had these conditions or if they had received a doctor’s diagnosis [17].

Mobility was assessed through self-reporting, with respondents indicating whether they encountered difficulties in climbing several flights of stairs, getting up from a chair, or dressing (including shoes and socks). Frailty was assessed by inquiring if respondents had been bothered by specific health conditions listed on a card for at least the past six months, with response options including “falling” and “fear of falling” [17].

The two inclusion criteria were the HGS above the gender-specific threshold at baseline (wave 5) and the availability of follow-up information on HGS from at least one of the subsequent waves (6, 7, or 8). On average, there was an interval of 5 years and 1 month between the first (WAVE 5) and the last HGS measurement.

In waves 5–8, the share of respondents who did not complete the HGS test were approximately 9–10%. An analysis of SHARE respondents showed that the absence of HGS information was not random, as the ability to perform the test decreases with advancing age [18]. The measurement of HGS is physically demanding, leading to relatively high number of missing information with age. Respondents with very low HGS presumably had a systematically higher probability to refuse or an inability to perform the test. Therefore, the prevalence of low HGS is probably underestimated in the SHARE sample. For quality of life and Euro-D scores, a category of “missing” was added, which prevented further shrinkage of the sample due to “don’t know” and “refuse” responses. The missing category was assigned to all individuals, who did not provide valid information for the respective question. For all other covariates, cases with missing information were excluded from the analysis sample.

### Statistical analysis

To identify personal characteristics influencing the risk of future low HGS, multiple regression analyses were employed. Given the dichotomous nature of the indicator for low HGS (present or absent), a logit regression model was utilized, as described by the following equation:

$$\text{ln}\left(\frac{\pi }{1-\pi }\right)={\beta }_{0}+{\beta }_{1}{X}_{1}+{\beta }_{2}{X}_{2}+\dots +{\beta }_{k}{X}_{k}+\epsilon$$

Here, $\pi$ represents the risk of low HGS, and $\pi /(1-\pi )$ represents the odds of low HGS. The variables ${X}_{1}, {X}_{2},\dots ,{X}_{k}$ represent potential personal characteristics influencing low HGS risk, while ${\beta }_{1},{\beta }_{2},\dots ,{\beta }_{k}$ denote the corresponding effects. $\epsilon$ accounts for the error term. Estimated parameters are presented as odds ratios, where values close to 1 suggested a minimal impact on low HGS risk, values significantly above 1 indicated an increased risk, and values below 1 suggested a reduced risk. Lastly, we added the variable effects as percentage points change for calculating the risk of low HGS (fractions). Statistical analyses were performed using STATA software package version 18.0 (StataCorp LLC, College Station, TX).

## Results

We found a slightly higher incidence of low HGS among men (7.38%) than in women (6.97%) (Table 1). Conversely, the participants with normal HGS exhibited a 7.2% risk of developing low HGS within the next six years. The proportions of participants with low HGS progressively increased with advancing age. For example, among the participants aged 50–59, the occurrence of low HGS was found in 1.75% of participants. Conversely, 37.53% of participants aged 90 or above exhibited a low HGS (Table 1).

**Table 1:** Basic characteristics of the study population with low handgrip strength (HGS) according to the criteria set by the European Working Group for Sarcopenia in Older Adults (HGS; men ≤ 27 kg, women ≤ 16 kg)

		Control	Low HGS	Low HGS	P-value
		Count	Count	%	
All		39,167	3016	7.15	
Gender	Male	17,515	1395	7.38	0.102
	Female	21,652	1621	6.97	
Age	50–60	5175	92	1.75	0.000
	60–69	15,528	413	2.59	
	70–79	12,922	922	6.66	
	80–89	4986	1255	20.11	
	90 +	556	334	37.53	
Quality of life	12–24	540	94	14.83	0.000
	25–36	11,279	1292	10.28	
	37–48	26,195	1482	5.35	
	Missing	1153	148	11.38	
Euro depression scale	0	9528	493	4.92	0.000
	1–3	20,957	1521	6.77	
	4–6	6949	755	9.80	
	7–12	1443	205	12.44	
	Missing	290	42	12.65	
Difficulty with climbing several flights of stairs	No	31,718	1767	5.28	0.000
	Yes	7449	1249	14.36	
Difficulty with getting up from chair	No	33,612	2187	6.11	0.000
	Yes	5555	829	12.99	
Difficulty with dressing one-self	No	37,472	2707	6.74	0.000
	Yes	1695	309	15.42	
Bothered by frailty, falling down	No	37,198	2655	6.66	0.000
	Yes	1969	361	15.49	
Bothered by frailty, fear of falling down	No	35,936	2352	6.14	0.000
	Yes	3231	664	17.05	
High blood pressure	No	24,376	1583	6.10	0.000
	Yes	14,791	1433	8.83	
High blood cholesterol	No	30,295	2262	6.95	0.003
	Yes	8872	754	7.83	
Diabetes or high blood sugar	No	34,994	2462	6.57	0.000
	Yes	4173	554	11.72	
Cancer	No	37,248	2847	7.10	0.086
	Yes	1919	169	8.09	
Alzheimer’s disease	No	39,003	2967	7.07	0.000
	Yes	164	49	23.00	
Stroke	No	38,098	2837	6.93	0.000
	Yes	1069	179	14.34	
Osteoarthritis	No	32,456	2290	6.59	0.000
	Yes	6711	726	9.76	

We found an inverse association between CASP-12 scores and the proportions of participants with low HGS. For example, among participants with CASP-12 scores of 12–24, 14.83% participants exhibited low HGS. Conversely, only 5.35% of participants with CASP scores of 37–48 exhibited low HGS (Table 1).

Next, we investigated the associations of Euro-D scores with HGS in the study population. We found that higher scores on Euro-D scale were associated with lower HGS. For example, among the participants with Euro-D scores of zero, the occurrence of low HGS was found in 4.92% participants. Conversely, Euro-D scores of 7–12 were associated with a low HGS in 12.44% of the participants (Table 1).

The difficulties with climbing several flights of stairs, getting up from a chair, or dressing were associated with a higher occurrence of low HGS. For example, the occurrence of low HGS was from 13–15.4% in participants with these difficulties compared to 6.1–6.7% in participants without these difficulties. We also found a similar association of low HGS with being bothered by frailty (falling down or fear of falling down) among the study participants. Thus, the occurrence of low HGS was found in 15.5–17% of participants who were bothered by frailty. Conversely, only 6.1–6.7% of participants not bothered by frailty exhibited low HGS (Table 1).

Among comorbidities, the presence of diabetes mellitus, high blood pressure, cancer, Alzheimer’s disease, stroke, or osteoarthritis were associated with a higher occurrence of low HGS. Thus, the proportions of participants with low HGS were 9.8–23% in participants with these comorbidities, but only 6.6–7.1% in participants without these comorbidities (Table 1).

Next, we statistically adjusted for confounding factors before investigating the associations of various variables with low HGS (Table 2). We found that female gender was associated with an approximately 1%-point lower incidence rate of low HGS than males. An advancing age also appeared as a significant predictor of low HGS. For example, the participants aged 90 or above exhibited 48%-points higher incidence of low HGS than participants aged 50–59 years (Table 2).

**Table 2:** Regression model for the risk of developing low handgrip strength (HGS) according to the criteria set by the European Working Group for Sarcopenia in Older Adults (HGS; men ≤ 27 kg, women ≤ 16 kg) during 2012–2018 given the baseline characteristics in 2012 among the European older adults

		Odds ratio		Fraction	
		Full	Significant	Full	Significant
Female		0.807***	0.803***	−0.00951***	−0.00974***
Age	60–69	1.421**	1.415**	0.0161**	0.0158**
	70–79	3.565***	3.517***	0.0707***	0.0698***
	80–89	11.22***	11.07***	0.241***	0.239***
	90+	24.74***	24.51***	0.483***	0.481***
Quality of life	25–36	0.895		−0.00477	
	37–48	0.638***	0.713***	−0.0211**	−0.0156***
	Missing	0.856		−0.00636	
Euro depression scale	1–3	1.135*	1.135*	0.00552*	0.00553*
	4–6	1.323***	1.337***	0.0133***	0.0139***
	7–12	1.500***	1.553***	0.0211**	0.0233***
	Missing	1.515*	1.493*	0.0219	0.0211
Difficulty with climbing several flights of stairs		1.503***	1.510***	0.0200***	0.0202***
Difficulty with getting up from chair		1.149**	1.168**	0.00634*	0.00713**
Difficulty with dressing one-self		1.126		0.00547	
Bothered by frailty, falling down		1.204**	1.214**	0.00875*	0.00921*
Bothered by frailty, fear of falling down		1.369***	1.385***	0.0155***	0.0161***
High blood pressure		0.940		−0.00269	
High blood cholesterol		0.907*	0.898*	−0.00417*	−0.00460*
Diabetes or high blood sugar		1.376***	1.367***	0.0156***	0.0153***
Cancer		0.862		−0.00611	
Alzheimer’s disease		1.389		0.0167	
Stroke		1.209*	1.218*	0.00900	0.00939*
Osteoarthritis		1.026		0.00112	
N		42,183	42,183	42,183	42,183

*p < 0.05, **p < 0.01, ***p < 0.001

We also found an inverse association between quality of life and low HGS. Thus, the highest scores on CAS-12 were associated with a 29% reduction in the occurrence of low HGS. Similarly, higher scores on Euro-D depression scales were associated with a 14–55% higher risk of developing low HGS, which corresponds to 0.6–2.3%-points higher incidence of HGS (Table 2).

Next, the difficulty climbing several flights of stairs was associated with a 2%-points higher incidence of developing low HGS. Similarly, difficulty getting up from a chair was associated with relatively modest but significantly 0.7%-points higher incidence of developing low HGS. Moreover, participants bothered by frailty had 0.9–1.6%-points higher incidence of developing low HGS compared to the participants not bothered by frailty (Table 2).

Among comorbidities, the presence of diabetes mellitus or stroke were associated with higher risks of developing low HGS (1.5%-points and 0.9%-points, respectively). However, similar effects were not found for other comorbidities, including cancer, Alzheimer’s disease, high blood pressure, and osteoarthritis (Table 2).

In general, advancing age was associated with a progressively higher proportion of participants with low HGS (Table 3). However, this trend was affected by the quality of life, diabetes mellitus, and stroke. Thus, the participants aged 50–59 with low CASP-12 scores, diabetes mellitus, or stroke had a higher proportion of low HGS than the participants aged 60–69. For other age groups, the occurrence of low HGS increased with advancing age irrespective of the comorbidities (Table 3).

**Table 3:** The incidence of low handgrip strength (HGS) according to the criteria set by the European Working Group for Sarcopenia in Older Adults (HGS; men ≤ 27 kg, women ≤ 16 kg) during 2012–2018 by age-groups among the European older adults

	Age, years:	50–59	60–69	70–79	80–89	90+	N
Gender	Male	1.6	2.3	6.7	21.7	39.7	18,910
	Female	1.9	2.8	6.7	18.7	35.9	23,273
Quality of life	12–24	7.8	7.2	16.6	29.1	36.4	634
	25–36	3.3	4.2	9.4	24.2	41.4	12,571
	37–48	1.0	1.7	5.2	17.1	34.7	27,677
	Missing	1.8	6.3	6.8	23.9	37.8	1301
Euro depression scale	0	0.7	1.6	4.1	17.9	36.1	10,021
	1–3	1.5	2.3	6.4	19.2	34.6	22,478
	4–6	3.4	3.9	9.5	22.9	43.9	7704
	7–12	3.1	6.3	13.6	28.4	50.0	1648
	Missing	2.5	7.2	12.5	22.4	31.3	332
Difficulty with climbing several flights of stairs	No	1.4	2.0	5.2	17.3	32.8	33,485
	Yes	4.5	5.6	11.6	25.3	43.0	8698
Difficulty with getting up from chair	No	1.5	2.2	5.9	18.4	34.8	35,799
	Yes	4.0	5.2	10.5	25.8	44.7	6384
Difficulty with dressing one-self	No	1.7	2.4	6.3	19.4	36.7	40,179
	Yes	4.1	6.7	12.9	28.4	45.2	2004
Bothered by frailty, falling down	No	1.7	2.4	6.4	19.2	36.6	39,853
	Yes	3.6	6.6	10.4	28.9	42.4	2330
Bothered by frailty, fear of falling down	No	1.6	2.2	6.1	18.4	34.5	38,288
	Yes	4.9	8.1	11.3	28.1	45.1	3895
High blood pressure	No	1.7	2.4	6.5	19.7	37.0	25,959
	Yes	1.8	3.1	6.9	20.5	38.1	16,224
High blood cholesterol	No	1.8	2.5	6.5	20.8	37.2	32,557
	Yes	1.7	3.1	7.2	18.2	38.9	9626
Diabetes or high blood sugar	No	1.6	2.4	5.9	19.3	38.3	37,456
	Yes	4.7	4.5	11.0	24.6	31.3	4727
Cancer	No	1.8	2.6	6.7	20.3	37.6	40,095
	Yes	1.3	3.2	6.4	17.9	36.8	2088
Alzheimer’s disease	No	1.7	2.6	6.6	20.0	37.3	41,970
	Yes	0.0	12.5	18.2	26.2	47.8	213
Stroke	No	1.7	2.5	6.5	19.8	37.4	40,935
	Yes	6.1	5.2	12.1	24.9	39.6	1248
Osteoarthritis	No	1.6	2.3	6.4	19.8	36.5	34,746
	Yes	2.6	4.2	7.9	21.2	40.4	7437
Sample size		5267	15,941	13,844	6241	890	42,183

We also found a generally consistent pattern of low HGS with high depression scores irrespective of other variables (Table 4). However, the participants from the youngest and oldest age groups, lowest and highest quality of life, or stroke exhibited a slight deviation from this pattern (Table 4).

**Table 4:** The incidence of low handgrip strength (HGS) according to the criteria set by the European Working Group for Sarcopenia in Older Adults (HGS; men ≤ 27 kg, women ≤ 16 kg) during 2012–2018 by the Euro Depression Scale among the European older adults

	Depression level	0	1–3	4–6	7–12	Missing	N
Gender	Male	5.3	7.6	10.3	11.7	12.5	18,910
	Female	4.4	6.1	9.6	12.7	12.8	23,273
Age	50–60	0.7	1.5	3.4	3.1	2.5	5267
	60–69	1.6	2.3	3.9	6.3	7.2	15,941
	70–79	4.1	6.4	9.5	13.6	12.5	13,844
	80–89	17.9	19.2	22.9	28.4	22.4	6241
	90 +	36.1	34.6	43.9	50.0	31.3	890
Quality of life	12–24	0.0	15.9	16.6	13.3	25.0	634
	25–36	8.0	9.4	11.6	13.0	9.0	12,571
	37–48	4.4	5.6	6.7	6.4	7.9	27,677
	Missing	6.9	9.3	13.6	19.4	20.2	1301
Difficulty with climbing several flights of stairs	No	4.0	5.2	7.0	8.7	10.5	33,485
	Yes	13.5	13.7	15.1	16.2	17.5	8698
Difficulty with getting up from chair	No	4.5	5.9	8.5	9.4	10.7	35,799
	Yes	11.2	12.0	13.3	17.3	19.0	6384
Difficulty with dressing one-self	No	4.8	6.4	9.2	11.0	13.2	40,179
	Yes	12.7	14.9	15.3	19.4	7.1	2004
Bothered by frailty, falling down	No	4.8	6.4	9.2	10.7	12.3	39,853
	Yes	10.6	15.0	15.5	21.7	16.7	2330
Bothered by frailty, fear of falling down	No	4.6	5.9	8.2	9.4	12.2	38,288
	Yes	14.5	16.6	17.1	20.7	14.8	3895
High blood pressure	No	4.4	5.9	8.7	9.4	9.3	25,959
	Yes	6.0	8.2	11.3	15.8	16.8	16,224
High blood cholesterol	No	4.8	6.6	9.9	12.0	10.4	32,557
	Yes	5.3	7.3	9.6	13.4	19.5	9626
Diabetes or high blood sugar	No	4.7	6.1	9.1	11.6	12.2	37,456
	Yes	6.8	11.8	14.1	16.1	15.6	4727
Cancer	No	4.9	6.7	9.9	12.7	12.7	40,095
	Yes	6.5	7.8	9.0	9.8	12.5	2088
Alzheimer’s disease	No	4.9	6.7	9.7	12.1	12.7	41,970
	Yes	20.0	20.5	24.4	31.0	12.5	213
Stroke	No	4.8	6.6	9.5	12.5	12.3	40,935
	Yes	14.1	13.1	17.3	11.9	18.2	1248
Osteoarthritis	No	4.7	6.3	9.1	11.7	12.9	34,746
	Yes	6.8	8.9	11.9	13.9	11.5	7437
Sample size		10,021	22,478	7704	1648	332	42,183

After statistical adjustment, we found that men had a slightly higher occurrence of low HGS compared to women (Table 5). However, this observation was not consistent across all variables, as women had a higher occurrence of low HGS than men for approximately 30% of the variables (Table 5).

**Table 5:** The incidence of low handgrip strength (HGS) according to the criteria set by the European Working Group for Sarcopenia in Older Adults (HGS; men ≤ 27 kg, women ≤ 16 kg) during 2012–2018 by gender among the European older adults

	Gender	Males	Females	N
Age	50–60	1.6	1.9	5267
	60–69	2.3	2.8	15,941
	70–79	6.7	6.7	13,844
	80–89	21.7	18.7	6241
	90 +	39.7	35.9	890
Quality of life	12–24	14.3	15.1	634
	25–36	10.7	10.0	12,571
	37–48	5.7	5.1	27,677
	Missing	11.8	11.0	1301
Euro depression scale	0	5.3	4.4	10,021
	1–3	7.6	6.1	22,478
	4–6	10.3	9.6	7704
	7–12	11.7	12.7	1648
	Missing	12.5	12.8	332
Difficulty with climbing several flights of stairs	No	5.7	4.9	33,485
	Yes	16.1	13.4	8698
Difficulty with getting up from chair	No	6.5	5.8	35,799
	Yes	14.2	12.3	6384
Difficulty with dressing one-self	No	7.0	6.6	40,179
	Yes	15.0	15.8	2004
Bothered by frailty, falling down	No	7.0	6.4	39,853
	Yes	18.0	14.4	2330
Bothered by frailty, fear of falling down	No	6.7	5.7	38,288
	Yes	19.4	16.2	3895
High blood pressure	No	6.6	5.7	25,959
	Yes	8.6	9.0	16,224
High blood cholesterol	No	7.4	6.6	32,557
	Yes	7.3	8.3	9626
Diabetes or high blood sugar	No	6.9	6.3	37,456
	Yes	10.7	12.8	4727
Cancer	No	7.3	7.0	40,095
	Yes	9.7	6.8	2088
Alzheimer’s disease	No	7.3	6.9	41,970
	Yes	21.7	24.0	213
Stroke	No	7.1	6.8	40,935
	Yes	14.0	14.7	1248
Osteoarthritis	No	7.0	6.3	34,746
	Yes	10.2	9.6	7437
Sample size		18,910	23,273	42,183

**Fig. 1:**
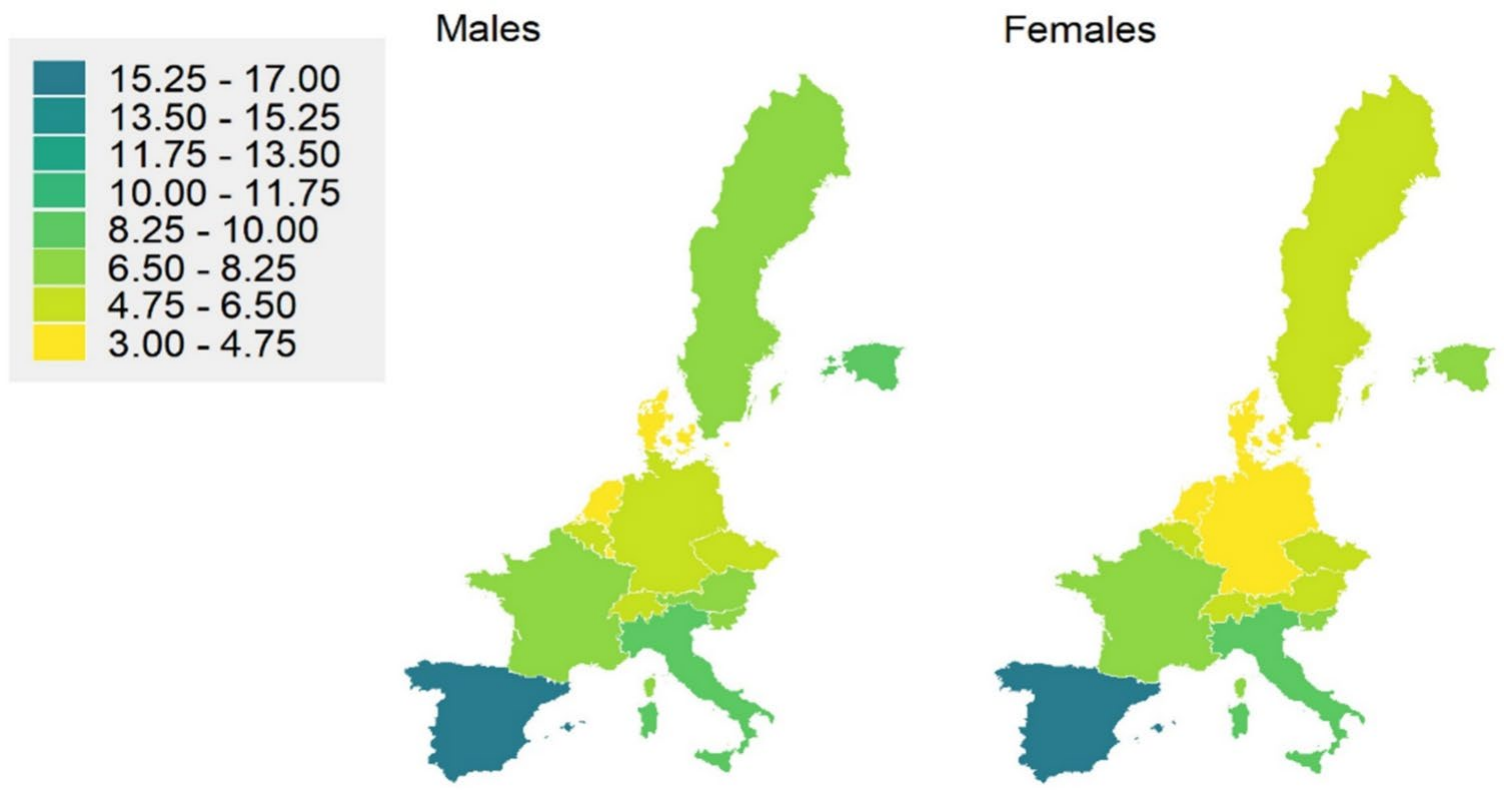
The proportion of European older adults aged 50 or above with low handgrip strength (HGS) (males; ≤ 27 kg, females; ≤ 16 kg) in European countries

Lastly, we investigated the occurrence of low HGS in different geographical regions of Europe (Fig. 1). We found a higher incidence of low HGS in the southern European countries. Specifically, Spain demonstrated an occurrence of low HGS among 15.9% of men and 15.7% of women (Fig. 1). Conversely, the lowest incidence of low HGS was found in central European countries. For example, the incidence of low HGS was found in only 3.8% of men and 3.9% of women in Denmark, and in 3.9% of men and 3.4% of women in Netherlands. Conversely, the remaining European countries demonstrated a varied distribution of participants with low HGS (Fig. 1).

## Discussion:

To our best knowledge, this is the first longitudinal study investigating the risk factors associated with developing a low HGS in a composite cohort from a whole continent. We found poor quality of life, diabetes mellitus, and stroke, as significant risk factors associated with the future development of low HGS. Moreover, we also observed that difficulties climbing several flights of stairs, getting up from a chair, frailty, and falling down significantly increased the risk of developing low HGS. Lastly, male gender and advancing age were also associated with low HGS.

Despite some inconsistent findings, the association between a poor quality of life and low HGS is generally recognized [19]. However, most relevant studies investigate the quality of life in patients with baseline poor HGS. Here, we show that poor quality of life at baseline can also contribute to developing low HGS in the future. The CASP-12 used here primarily evaluates the cognitive and emotional health of daily living associated with control, autonomy, pleasure, and self-realization [17]. Conversely, physical performance is not the primary focus of CASP-12. We have previously reported the correlation between CASP-12 scores and HGS in a cross-sectional observation of European older adults [17]. Here, we expand these findings to report that low CASP-12 can independently predict the future onset of low HSG in European older adults. This observation is consistent with the correlation between mental health and low HGS in older adults [12, 20]. In addition, it supports the well-established correlation of poor physical performance with low HGS and muscle weakness in old age [6, 21].

The robust association between depression and low HGS further strengthens the coupling of mental and physical health. A dose-dependent inverse association has been described between HGS and the risk of developing depressive symptoms in older adults [22]. This finding supports our observation that a progressive increase in Euro-D depression scores was associated with a progressively higher risk of developing low HGS. Together, these observations suggest a bidirectional crosstalk between depression and low HGS. Depression can cause muscle weakness through multiple mechanisms. For example, older adults with depression have higher levels of plasma cortisol [23], which is an independent risk factor for muscle weakness [24]. Depressive patients have a sedentary lifestyle with physical inactivity [25], which may contribute to low HGS [26]. Lastly, depressive patients also exhibit elevated systemic inflammation, which can cause myopathy and muscle weakness [27]. Together, these attributes may provide a causal association between depression and the future onset of low HGS.

Among comorbid conditions, diabetes mellitus, and stroke emerged as independent risk factors for low HGS. Several studies have characterized the associations of these conditions with reduced HGS [9, 28]. For example, dyslipidemia exhibits a robust inverse correlation with HGS after adjustment for other confounding factors [29]. Similarly, diabetic myopathy is a well-established occurrence in patients with prolonged diabetes mellitus and is characterized by an accelerated loss of muscle mass and strength [28]. These effects of diabetes mellitus on skeletal muscle are attributed to the muscle protein glycosylation, elevated oxidative stress, and dysregulated calcium handling [30]. Patients with a history of stroke also exhibited a higher risk of developing low HGS, possibly due to prolonged mechanical unloading of skeletal muscle and concomitant morbidities, such as hypertension and clotting defects [31].

As expected, patients with physical disabilities were at higher risk of developing low HGS. Specifically, we found that difficulties climbing several flights of stairs and getting up from a chair were significant risk factors for developing low HGS. These activities primarily require the functioning of lower limb muscles. A moderate concordance exists between HGS and lower limb muscle functions [32]. Thus, the patients with a low HGS exhibit lower strength of lower limb muscles. HGS also exhibits a weak association with postural balance [32], which is required for climbing stairs and rising from a chair. These findings support the interface of difficulties in climbing stairs and rising from a chair with future onset of low HGS.

We also found an association of advancing age with a higher risk of developing a low HGS. This is attributed to progressive muscle degeneration, physical inactivity, hormonal imbalance, and other factors associated with sarcopenia [33]. Interestingly, the prevalence of low HGS dramatically increased from the ninth decade of life onward. A rapid decline of muscle strength and a higher occurrence of sarcopenia is reported after the eighth decade of life independent of the diagnostic criteria and low HGS and sarcopenia [8]. The progressively cumulative effects of the causative factors of sarcopenia may account for these findings. Among genders, the prevalence of low HGS was slightly higher in men than in women. Previous studies are inconsistent about the gender-specific comparison of the prevalence of sarcopenia and low HGS in older adults [34]. This may partly be due to various diagnostic criteria of sarcopenia and low HGS. For example, HGS cutoff values of 20 and 17 kg reveal different prevalence of sarcopenia among women than in men [34]. We used an HGS cutoff value of 16 kg for women, which revealed slightly lower prevalence of low HGS among women than in men. This observation is consistent with a lower prevalence of sarcopenia among European women, when using a similar cutoff value for HGS [34].

This study has several strengths. SHARE is a validated and internationally standardized dataset. We used the HGS cutoff value of 16 kg, which is relevant to European population according to the criteria set by the EWGSOP2 [6]. The longitudinal study design builds our confidence in the associations of risk factors with HGS. A large sample size from various European regions homogenizes the potential effects of socioeconomics, genetic, and racial factors. However, this study has some limitations. We did not measure the status of hormone replacement therapy in postmenopausal women, which can affect the HGS due to its anabolic actions. We did not measure physical activities of the participants, which can independently affect skeletal muscle health and HGS.

The findings from this study hold several practical applications. Prediction and monitoring of a low HGS can provide a comprehensive health assessment in domestic and clinical settings before more rigorous health assessment tools are implemented. A low HGS may also be useful for early disease detection in subclinical stages, warranting further evaluation of patients with low HGS. The serial measurements of HGS performed in this study can be useful for monitoring of generalized health and specific diseases. HGS evaluation also provides a cost-effective, user-friendly, and non-invasive tool for health assessment, that can be implemented in most domestic settings. Lastly, based on already established normative values of HGS for genders, age groups, and geographical regions, our findings are applicable for diverse populations across the globe.

Collectively, we report several risk factors associated with the future development of low HGS. Specifically, we found male gender, advancing age, poor quality of life, diabetes mellitus, stroke, and physical disabilities as significant risk factors for low HGS. Several of these risk factors can be evaluated in domestic settings and may help identify high-risk patients for clinical assessment. Our findings may be relevant for clinicians and policymakers for identifying older adults with muscle weakness.

## Data Availability

The data is publicly available after application from https://share-eric.eu/. The access to data requires an individual free registration followed by the acceptance of the SHARE Conditions and signing the SHARE User Statement. After acceptance of these documents, data can be downloaded using the personal ID and password.
